# Exploration of the hypoglycemic mechanism of Fuzhuan brick tea based on integrating global metabolomics and network pharmacology analysis

**DOI:** 10.3389/fmolb.2023.1266156

**Published:** 2024-01-18

**Authors:** Xingliang Xiang, Shanqin You, Zhaoxiang Zeng, Jinlin Xu, Yuqi Lin, Yukun Liu, Lijun Zhang, Rongzeng Huang, Chengwu Song, Shuna Jin

**Affiliations:** ^1^ School of Pharmacy, Hubei University of Chinese Medicine, Wuhan, Hubei, China; ^2^ School of Life and Health Sciences, Hainan University, Haikou, Hainan, China; ^3^ Department of Pharmacy, Ezhou Central Hospital, Ezhou, Hubei, China; ^4^ School of Basic Medical Sciences, Hubei University of Chinese Medicine, Wuhan, Hubei, China; ^5^ Hubei Shizhen Laboratory, Wuhan, Hubei, China

**Keywords:** diabetes mellitus, Fuzhuan brick tea, metabolomics, network pharmacology, LC-MS/MS

## Abstract

**Introduction:** Fuzhuan brick tea (FBT) is a worldwide popular beverage which has the appreciable potential in regulating glycometabolism. However, the reports on the hypoglycemic mechanism of FBT remain limited.

**Methods:** In this study, the hypoglycemic effect of FBT was evaluated in a pharmacological experiment based on Kunming mice. Global metabolomics and network pharmacology were combined to discover the potential target metabolites and genes. In addition, the real-time quantitative polymerase chain reaction (RT-qPCR) analysis was performed for verification.

**Results:** Seven potential target metabolites and six potential target genes were screened using the integrated approach. After RT-qPCR analysis, it was found that the mRNA expression of VEGFA, KDR, MAPK14, and PPARA showed significant differences between normal and diabetes mellitus mice, with a retracement after FBT treatment.

**Conclusion:** These results indicated that the hypoglycemic effect of FBT was associated with its anti-inflammatory activities and regulation of lipid metabolism disorders. The exploration of the hypoglycemic mechanism of FBT would be meaningful for its further application and development.

## 1 Introduction

Type 2 diabetes mellitus (T2DM) has evolved into the majority of diabetes mellitus, with characteristic symptoms of relative insulin deficiency and chronic hyperglycemia (Mao et al., 2021). In recent decades, T2DM has become a worldwide public health burden due to modern lifestyles (Collaboration, 2016). Obesity caused by a high-caloric diet and a lack of exercise is one of the strongest risk factors (Kusminski et al., 2016). With the development of T2DM, patients have to suffer from profound psychological and physical distress caused by numerous complications (Hackett and Steptoe, 2017; Zhou et al., 2017). In the lengthy treatment process, some effective diet therapy methods would be receptive and easy to perform. Consequently, the exploration of therapies or prevention schemes based on widely accepted foods would be a necessity (Evert et al., 2019).

As one of three major beverages (Peng et al., 2016; Yu et al., 2020), tea (*Camellia sinensis*) is closely related to the lifestyles and dietary habits of people in many countries (Roy et al., 2008; Soh et al., 2017; Tsuboi et al., 2019; Inoue-Choi et al., 2022). Fuzhuan brick tea (FBT), as a Chinese traditional tea, belongs to dark tea with a unique fermentation process. In the fermentation procedures, many special sensory characteristics and health benefits of FBT were produced after being fermented by the “golden flower fungus” (*Aspergillus cristatus*) (Xu et al., 2011). In ancient China, FBT was not only a tasty beverage but also a specific medicinal plant. Accumulating evidence has also indicated that FBT is a functional beverage with many bioactivities (Chen et al., 2018; Du et al., 2019; Jing et al., 2020; Zhou et al., 2021). Moreover, in our previous research studies, it was discovered that FBT could regulate the levels of blood glucose in T2DM mice (Xiang et al., 2020), which also showed the inhibitory effect of α-glucosidase *in vitro* (Xiang et al., 2021). Therefore, as a popular beverage with potential hypoglycemic activity, a comprehensive investigation on the therapeutic effect and mechanism is necessary for the further development and application of FBT.

With the development of instrumentation such as mass spectrometry (MS), metabolomics analyses could benefit from these high-dimensional biological data. Due to the unique advantage of its integrality and dynamic conditions, global metabolomics has become a comprehensive and efficient strategy for studying the interactions between variation in endogenous supersession and the exogenous intervention of disease or treatment (Warth et al., 2017; Meng et al., 2022b). Meanwhile, network pharmacology could provide a series of systematic and comprehensive views by focusing on the interactions between “drug-target-gene-disease” (Zhang et al., 2019). Due to this advantage, network pharmacology has been a popular and efficient tool for explaining the mechanisms of complex medicines (Guo et al., 2022; He et al., 2022). As mentioned, global metabolomics analysis could explore metabolic information based on experimental data. In addition, the network pharmacology strategy is adept in the predicting of action targets and pathways based on the network database. Hence, it is possible to holistically reveal the overall skeleton of biological processes by integrating global metabolomics and network pharmacology.

In this study, the hypoglycemic effect of FBT drinking was explored by a pharmacological experiment on Kunming mice with low aggression and strong adaptability, which were commonly used for T2DM research (Meng et al., 2022a). A comprehensive strategy integrating global metabolomics and network pharmacology was applied to investigate the potential action pathways and target genes. Then, the screened target genes were verified by real-time quantitative polymerase chain reaction (RT-qPCR) analysis. Through the above systematic analyses, the potential effective metabolites, genes, and pathways were confirmed.

## 2 Materials and methods

### 2.1 Chemicals and materials

FBT (Mogen Golden Flower Tea Technology Co., Ltd., Hunan Province, China) was identified at the Hubei University of Chinese Medicine. As a continuation of previous studies, the mass-spectrogram fingerprint of FBT was available in the previously published reports (Xiang et al., 2020; Xiang et al., 2021). High-performance liquid chromatography (HPLC)-grade acetonitrile and methanol were obtained from Fisher Scientific (Fair Lawn, NJ, United States). HPLC-grade isopropyl alcohol was obtained from Sinopharm Chemical Reagent Co., Ltd. (Beijing, China). Formic acid (≥98%) was purchased from Merck & Co., Inc. (United States). Fexofenadine hydrochloride (≥98%) and streptozotocin (STZ) were purchased from Yuanye Biotechnology Co., Ltd. (Shanghai, China). Cholesterol was purchased from Xin He Biotechnology Co., Ltd. (Jiangsu, China). Cholate was purchased from Long De Biotechnology Co., Ltd. (Guangzhou, China). For the RNA extraction procedure, the SweScript RT II First-Strand cDNA Synthesis Kit (with gDNA remover) and Universal Blue SYBR Green qPCR Master Mix were purchased from Wuhan Servicebio Technology Co., Ltd. (Wuhan, China). Deionized water was produced using a Milli-Q water system (Millipore, Bedford, MA, United States). The basic diet of mice was purchased from HFK Biotechnology Co., Ltd. (Beijing, China), and its compositional data are shown in Supplementary Figure S1.

### 2.2 Experimental animals

Forty male Kunming mice (8 weeks old, weighing from 18 to 22 g, Beijing Vital River Laboratory Animal Technology Co., Ltd., Beijing, China) were selected for the experiment. The experimental environment was kept at 19°C–23°C. The light/dark cycle was set at 12/12 h. An acclimation period of 5 days was set before the initiation of experimentation. The animal experiment was approved by the Animal Ethics Committee of Hubei University of Chinese Medicine. Experimental animals were randomly divided into normal and diabetic mice groups, including one normal group (group N) and four diabetic groups (diabetic model group, group D; positive group, group *p*; high-dose drinking treatment group, group H; and low-dose drinking treatment group, group L). For responding to the depletion of numbers caused by accidental death and failed modeling, eight mice were first prepared for each group. The normal group was fed a basic diet (11.2% kcal from fat, 17.3% kcal from protein, and 71.5% kcal from carbohydrates) in the whole experiment. The four diabetic groups were fed a high-fat diet in the first 4 weeks and a basic diet in the later 4 weeks, as depicted in Supplementary Figure S2. The data on the basic diet and home-made high-fat diet are shown in Supplementary Tables S1, S2.

The establishing process of the diabetic mouse model is shown in Supplementary Figure S2. For the establishment of the diabetic mouse model, a high-fat diet was continued for 4 weeks as the base. Then, mice were injected twice with STZ (100 mg kg^−1^) intraperitoneally. STZ was dissolved in the citrate buffer (pH 4.2–5.0). A fasting blood glucose level higher than 6.1 mmol L^−1^ was considered the T2DM model. The success rate of the model establishment is higher than 80%.

In the 4-week treatment phase, the mice in group *p* were intragastrically administered with the metformin solution (0.4 mL, 40 mg kg^−1^·day^−1^). In the groups H and L, mice were provided with FBT decoction as a replacement of water, which they consumed freely. For the preparation of decoction, FBT powder was boiled in 1 L of deionized water for 2 h. The supernatant was collected and replenished to 1 L. The concentrations of FBT decoction were converted by human intake (high-dose, 10 g L^−1^; low-dose, 5 g L^−1^), and FBT decoction was refreshed daily. Intake of food and water (once daily), weight (twice weekly), and the fasting blood glucose level (each Sunday, 5th to 8th week) were recorded. Blood glucose measurement was done using the portable blood glucose meter (Sinocare Biosensing Co., Ltd., Changsha, China) by minimally invasive blood collection from the tail vein after fasting for 8 h. Finally, in harvest, ophthalmectomy after anesthetization was performed to collect blood and liver samples.

### 2.3 Global metabolomic analysis

#### 2.3.1 Preparation of samples

The serum samples were extracted using our previous method with a little modification (Shi et al., 2019). A measure of 40 μL of serum was mixed with 160 μL of acetonitrile and 50 μL of the internal standard solution (fexofenadine, 500 ng mL^−1^), vortexed for 2 min, and then stewed for 10 min in a refrigerator at 4°C. The mixed sample was centrifuged (12,830×*g*, 5 min) for collecting 100 μL of the supernatant, and then, it was filtered using microporous filters (0.22 μm) for impurity removal. All serum samples were collected using the above approach. A control sample was mixed with 20 μL of each sample for follow-up qualitative analysis and quality control (QC).

#### 2.3.2 LC-MS/MS conditions

An ACQUITY UPLC M-Class system coupled with a Waters Xevo G2-XS QTof System (Waters, Massachusetts, United States) was used to conduct this analysis. The chromatographic separation was supported using a Welch UPLC C18 column (100 × 2.1 mm, 1.8 μm). The injection volume and flow rate were set at 2 μL and 0.3 mL min^−1^, respectively. Mobile phases A and B were water/formic acid (1000:1, v/v) and methanol. The following binary gradient with linear interpolation was used: 0 min, 10% B; 15.0 min, 95% B; 20.0 min, 95% B; 21.0 min, 10% B; and 25.0 min, 10% B.

Data acquisition was performed in an ESI source. Both positive and negative ion modes were acquired for different purposes. The positive data were mainly for metabolomic analysis, and the negative data were mainly for confirming the structure of metabolites. The MS operating conditions were as follows: desolvation temperature, 500°C; source temperature, 100°C; desolvation gas flow, 600 L h^−1^; cone gas flow, 50 L h^−1^; capillary voltage, 3 kV; and cone voltage, 60 V. Raw data were acquired in the MS^E^ continuum mode in mass ranges of *m/z* 100–1200 and a scan duration of 0.5 s. The lock mass standard selected leucine enkephalin (500 pg mL^−1^).

#### 2.3.3 Data analysis

The mass information on metabolites was mainly referred to the Human Metabolome Database (https://www.hmdb.ca) and the METLIN database (http://metlin.scripps.edu). MassLynx V4.1 software (Waters, Massachusetts, United States) was used for raw data reading. For a data table to global metabolomic analysis, the data were processed using MarkerLynx XS (Waters, Massachusetts, United States), including peak extraction, peak alignment, and isotope peak exclusion. The mass tolerance and noise elimination level were set at 0.01 Da and 50, respectively. The false discovery rate (FDR) was used for multiple corrections.

For improving accuracy and applicability of data, two filters were set to preliminary exclude obvious interference terms: (1) features with retention time (RT) less than 2 min or more than 20 min were excluded according to the experimental elution gradient and (2) features with a detection rate less than 50% in the single group. Meanwhile, raw data were processed by a common logarithmic transformation. Missing values were replaced by half of the minimum intensity.

### 2.4 Network pharmacology analysis

#### 2.4.1 Analytical database and software

Chemical components of FBT were confirmed in our previous study (Xiang et al., 2020). The SwissTargetPrediction database (http://www.swisstargetprediction.ch/) was used to predict the relevant target proteins based on similarities in their structure with drugs. The GeneCards database (https://www.genecards.org/) was used to collect the associated target proteins of T2DM. The STITCH database (http://stitch.embl.de/) was used for searching the target proteins of differential metabolites. The STRING database (https://cn.string-db.org/) was used for establishing the protein–protein interaction (PPI) network. Gene Ontology (GO) function enrichment analyses and Kyoto Encyclopedia of Genes and Genomes (KEGG) pathway enrichment analyses were performed on the Metascape database (https://metascape.org/). Visualization and analysis of a component–metabolite–target–pathway–disease network were performed using Cytoscape 3.9.0 software (https://cytoscape.org/) based on overlapping target proteins of active components, critical biomarkers, and disease-related genes.

#### 2.4.2 Combination of network pharmacology and global metabolomics

The chemical compositions of FBT were confirmed based on previous studies (Xiang et al., 2020); the direct targets were searched from the SwissTargetPrediction database. The corresponding targets of T2DM were searched from the GeneCards database using the keyword “type II diabetes mellitus,” with the screening condition of “relevance score > 20”. In addition, the component–disease PPI network was analyzed by importing Cytoscape software; the screening condition of critical targets was set as “degree > 11, betweenness centrality > 0.00533, and closeness centrality > 0.5236.”

At first, the PPI network was established on the basis of FBT information (correlative target genes of components) and disease information (correlative target genes of T2DM), which discovered the component–disease critical targets. Then, the retrieval was performed based on metabolic information. Furthermore, the interaction network analysis integrated metabolites, correlative targets, and component–-disease PPI critical targets. Finally, the potential target genes with critical impacts on treatment were screened out.

### 2.5 RT-qPCR conditions

RT-qPCR based on the SYBR Green method was performed on a Bio-Rad CFX96 Real-time System (Hercules, CA). The total RNA sample was extracted from liver tissues using the RNA extraction solution. First-strand cDNA synthesis was performed using the SweScript RT II First Strand cDNA Synthesis Kit. The primer sequences are depicted in Supplementary Table S3. β-Actin was used for normalization of RT-qPCR results. The data were analyzed using the 2^-△△Ct^ method.

### 2.6 Statistical analysis

The statistical analyses were performed using SPSS 23.0 software (SPSS Inc., Chicago, IL, United States). The graph analyses were constructed using GraphPad Prism 7.0 (GraphPad Software Inc., San Diego, CA). Data were expressed as the mean ± SD. A *p*-value < 0.05 was considered statistically significant. The multivariate statistics analyses were performed using SIMCA 14.1 (Umetrics AB, Umeå, Sweden), including principal component analysis (PCA) and orthogonal partial least squares discriminant analysis (OPLS-DA).

## 3 Results

### 3.1 Effects of FBT on physiological indexes of diabetic mice

**FIGURE 1:**
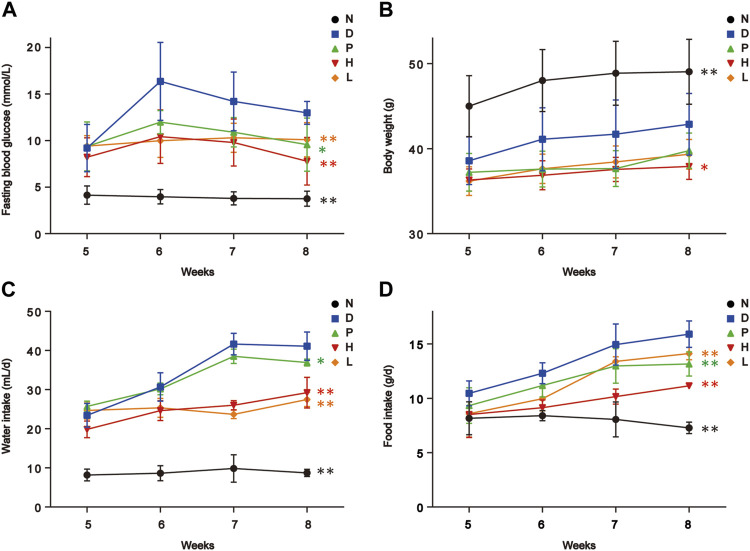
Biochemical parameters of mice in five groups. **(A)** Fasting blood glucose level. **(B)** Body weight. **(C)** Water intake. **(D)** Food intake. N, normal group; D, diabetic model group; P, positive group; H, high-dose FBT group; L, low-dose FBT group. Raw *p* of the *U*-test was used for the significance of each group compared to group D. * indicated *p* < 0.05; ** indicated *p* < 0.01.

After establishment of the diabetic model, the STZ-induced diabetic mice showed characteristic symptoms of T2DM, including polyphagia, polydipsia, polyuria, and body weight loss. In addition, through 4-week treatment, these conditions of diabetic mice had been improved, especially group H. Noteworthily, a prevalent decrease in fasting blood glucose levels happened in all diabetic groups with varying degrees in 8th week, which might indicate the partial recovery of pancreatic β-cells damaged by STZ. As depicted in Figure 1, FBT drinking significantly regulated the fasting glucose levels, water, and food intake of diabetic mice. In addition, the high-dose FBT-drinking group showed a more effective treatment than the low-dose group.

### 3.2 Global metabolomic profiling

**FIGURE 2:**
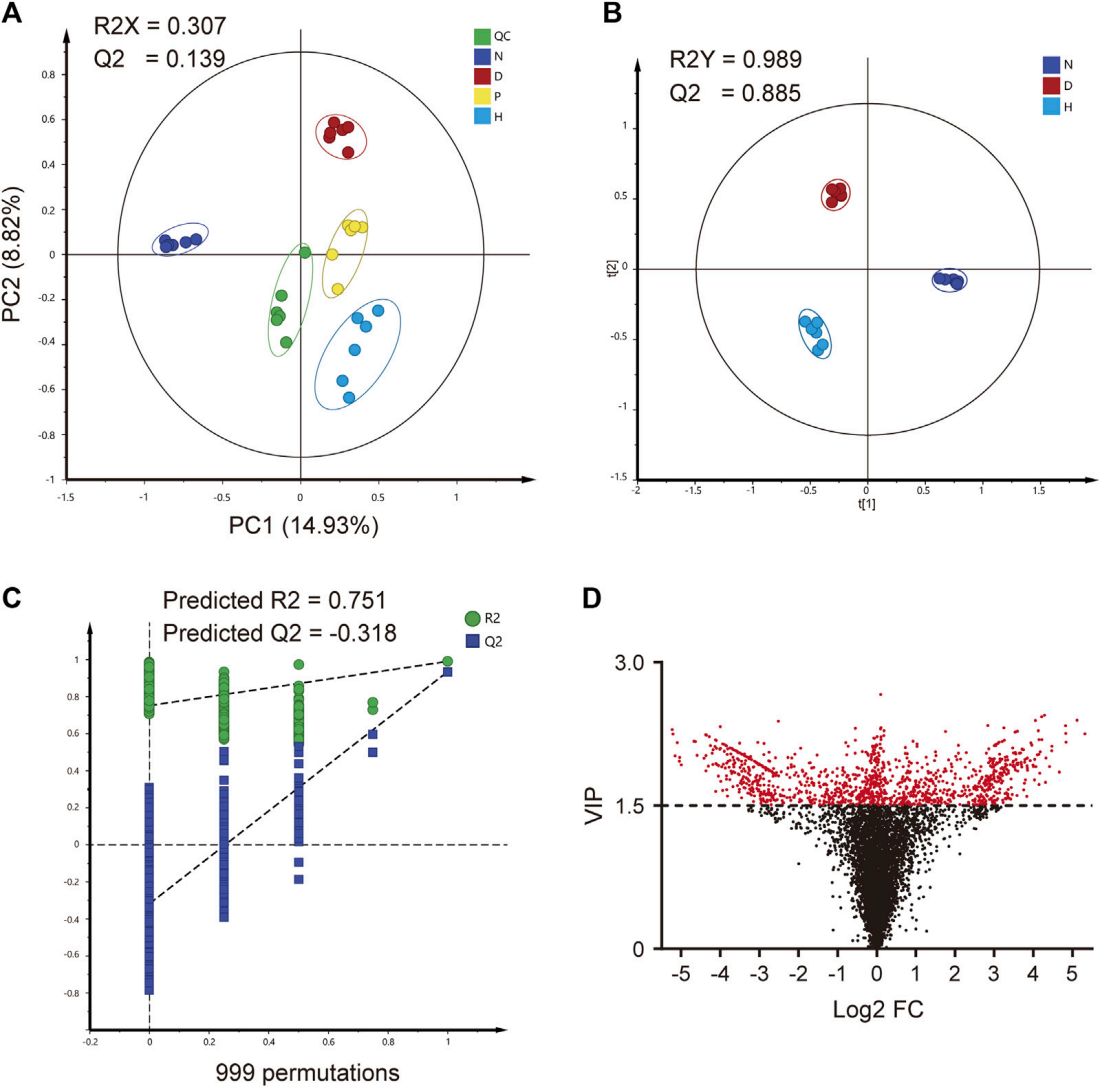
Global metabolomic profiling. **(A)** PCA analysis of four groups. **(B)** OPLS-DA analysis of three groups. **(C)** 999-times permutation test of the OPLS-DA model. **(D)** VIP value and log_2_ FC (fold change) of each feature. QC, quality control group; N, normal group; D, diabetic model group; P, positive group; H, high-dose FBT group.

The global metabolomic analysis was based on the MS information on serum samples. Due to the better therapeutic effect, group H was chosen as the representative FBT treatment group for subsequent analyses. At first, PCA was preliminarily applied to assess the differences between groups. Each group gathered at different places on the axis and tended to separate from each other, as depicted in Figure 2A. The result of the clustering analysis indicated that serum metabolite profiles in each group were significantly different. QC near the origin indicated the stability of the analytical methods and instruments.

For discovering discriminant features, OPLS-DA was applied to groups N, D, and H. As depicted in Figure 2B, the clustered situations also indicated the differences among the three groups. The fitness and reliability of model were excellent, according to the R2Y and Q2 values of 0.989 and 0.885, respectively. Furthermore, the 999-times permutation test also indicated its validity and predictability. The values of predicted R2 and Q2 from regression lines were 0.751 and −0.318, which were both smaller than those from actual models, as seen in Figure 2C. Meanwhile, the variable influence on projection (VIP) value of each feature was calculated, as depicted in Figure 2D. In addition, 1297 potential discriminant features were screened, while a VIP value greater than 1.5 was set as the threshold.

### 3.3 Discovery of discriminant metabolites

The identification of features was based on matching with their retention time, precursor ion information, and collision-induced dissociation (CID) fragmentation patterns. As a result, 31 metabolites were characterized, including phospholipids (PLs), lysophospholipids (LysoPLs), sphingomyelins (SMs), sphingosines (SPOs), fatty acid amides (FAMs), acylcarnitines (ACs), fatty acids (FAs), and steroid derivatives. For instance, C9 and C25 were identified as lysophosphatidylcholine (LysoPC) and SM by fragments at *m/z* 184.0733, 124.9999, and 104.1070. Among them, C9 was confirmed as SM due to its even-numbered nitrogen atoms. The component number and detailed information are depicted in Supplementary Table S4.

**FIGURE 3:**
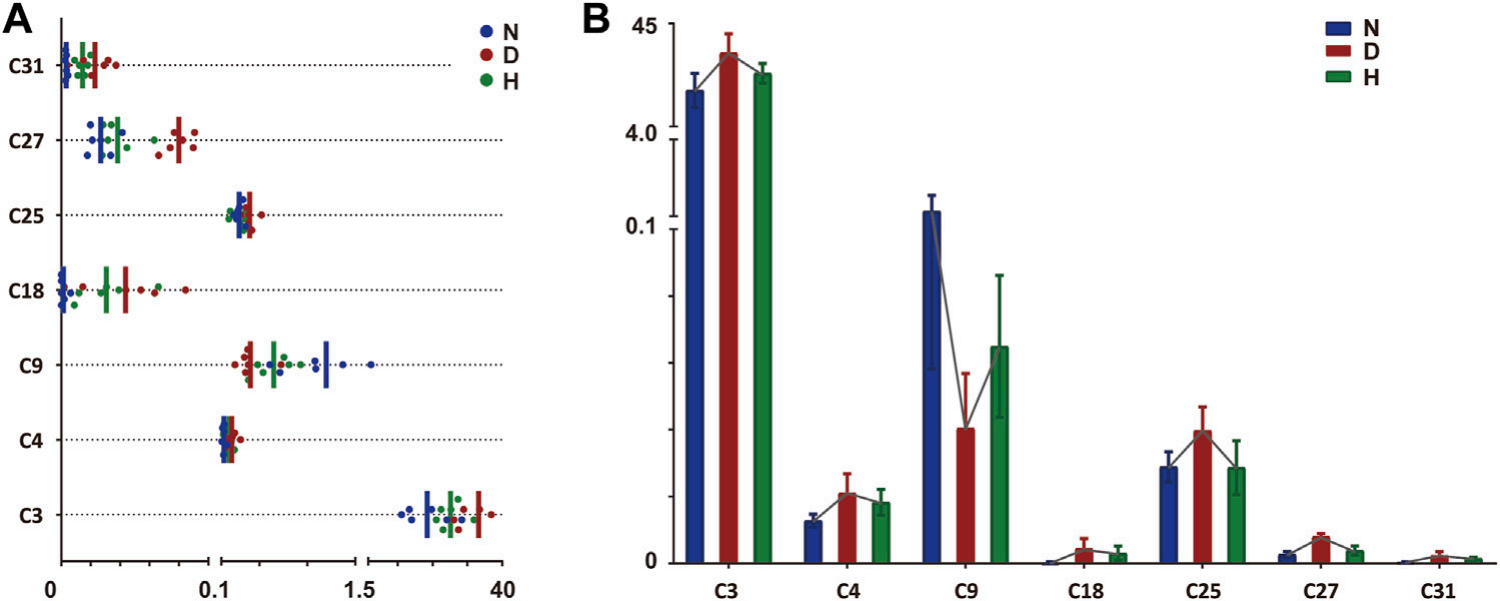
Relative quantitative analysis of seven discriminant metabolites in three groups. The value of each compound was log_10_-transformed. **(A)** Distribution of seven discriminant metabolites in different groups. Each point expresses one sample. Vertical line expresses the mean value of each group. **(B)** Relative contents of seven discriminant metabolites. Connected line of each metabolite indicates the changing trend. N, normal group; D, diabetic model group; H, high-dose FBT group.

The above-selected metabolites included potential targets recovered by FBT treatment, which should be further discovered. Two filters were set as the thresholds for the subsequent screening: (1) metabolites with significant differences (raw *p*-value < 0.05) between groups N and D; (2) metabolites with retracement after FBT drinking. There were 11 potential target metabolites remaining. Next, FDR correction was performed to adjust the *p*-value. Finally, a total of seven potential target metabolites (adjusted *p*-value < 0.05) remained, including FAM, AC, SM, LysoPC, long-chain fatty acid (LCFA), and steroid derivatives. The seven remained and excluded metabolites are shown in Table 1; Supplementary Table S5, respectively. The relative contents and changes of them in groups N, D, and H are displayed in Figure 3.

**TABLE 1:** MS and statistical information on seven potential target metabolites.

No.	RT (min)	Structure/name	Formula	Exact mass	Detected MS	MS/MS	VIP^a^	*U*-test^b^	FDR^c^	Fold change^d^	Recovery^e^	Direction of change^f^
C3	18.24	FAM (22:1)	C_22_H_43_NO	338.3423	[M+H]^+^ 338.3438	321.32, 303.31, 279.31, 237.26, 223.24, 209.23, 195.21, 181.20, 139.15, 123.12	2.153	0.010	0.040	1.804	Y	D
C4	17.97	AC (20:4)	C_27_H_45_NO_4_	448.3427	[M+H]^+^ 448.3419	336.22, 310.21, 296.18, 230.14, 144.10	2.118	0.010	0.040	1.649	Y	D
C9	17.46	SM (d17:1/20:3)	C_42_H_79_N_2_O_8_P	771.5652	[M+H]^+^ 771.5610	184.07, 125.00, 104.11	1.995	0.010	0.040	0.338	Y	U
C18	10.28	Trihydroxypregn-ene-dione	C_21_H_30_O_5_	363.2171	[M+H]^+^ 363.2205	303.19, 285.18, 261.19, 187.12, 163.11, 143.08	1.785	0.009	0.040	31.499	Y	D
C25	18.29	LysoPC (O-20:0)	C_28_H_60_NO_6_P	538.4237	[M+H]^+^ 538.4201	184.07, 124.10, 104.11	1.596	0.010	0.040	1.374	Y	D
C27	17.56	LCFA (20:2)	C_20_H_36_O_2_	309.2794	[M+H]^+^ 309.2800	263.27, 249.25, 207.21, 163.15, 139.15	1.558	0.004	0.040	2.994	Y	D
C31	10.19	Acetoxy-androstene-dione	C_21_H_28_O_4_	345.2066	[M+H]^+^ 345.2098	345.21, 317.20, 299.17, 285.18, 233.11, 217.09, 161.10, 123.08	1.520	0.006	0.040	6.953	Y	D

FAM, fatty acid amide; AC, acylcarnitine; SM, sphingomyelin; LysoPC, lysophosphatidylcholine; LCFA, long-chain fatty acid.

^a^ VIPs were calculated by establishing the OPLS-DA model with groups N, D, and H.

^b^Raw *p*-value of the *U*-test was used for the intensity of metabolites in groups N and D.

^c^ *p*-value was adjusted by false discovery rate (FDR) correction.

^d^ Fold change was the ratio of the intensity of metabolites in groups D and N.

^e^ Recovery trend of the relative content of the metabolite after FBT drinking. Y indicated recovering after FBT drinking.

^f^ Direction of change after FBT drinking. U indicated upregulation; D indicated downregulation.

### 3.4 Network pharmacology analysis combined with discriminant metabolites

**FIGURE 4:**
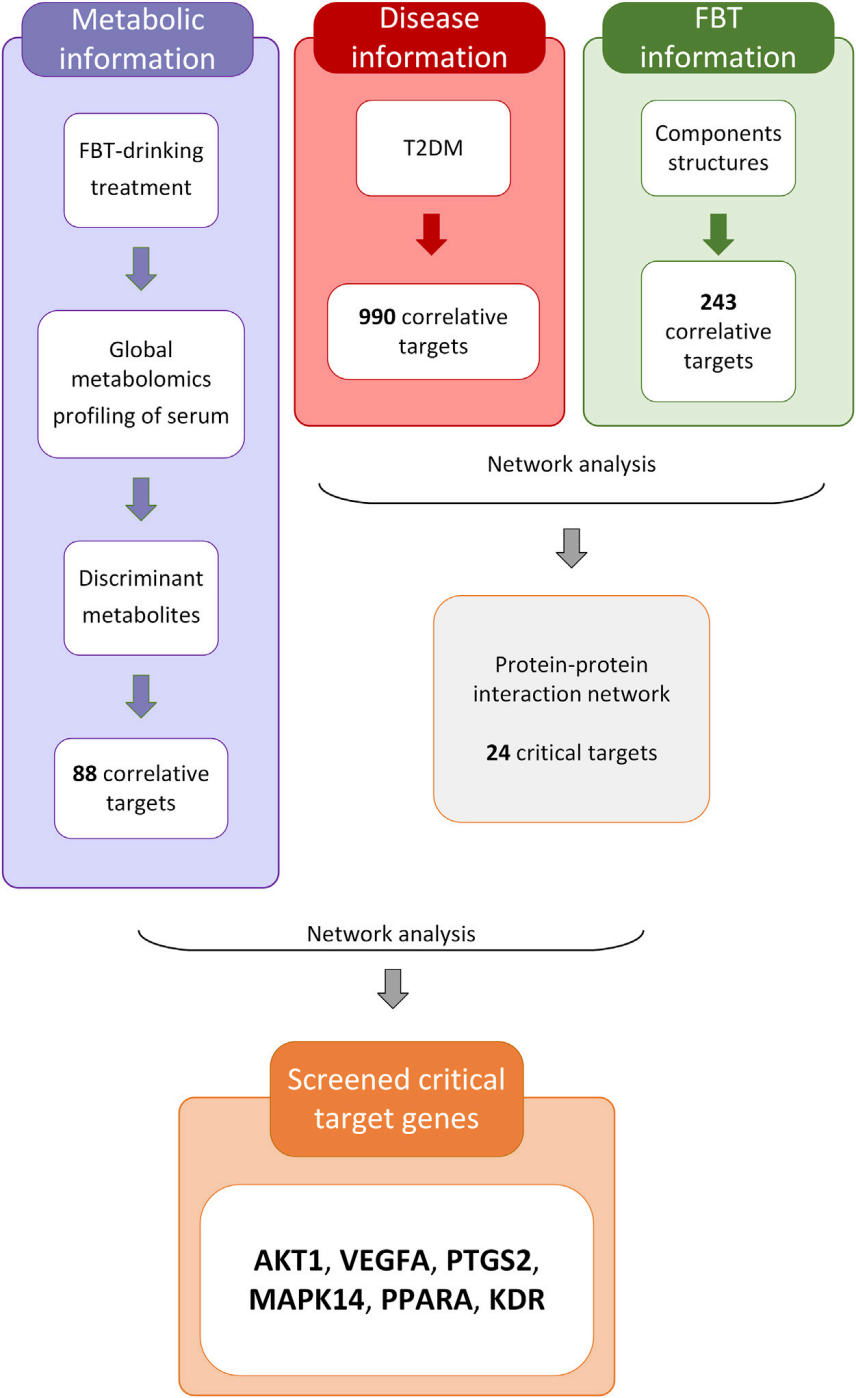
Analytical process integrating network pharmacology and global metabolomics.

**FIGURE 5:**
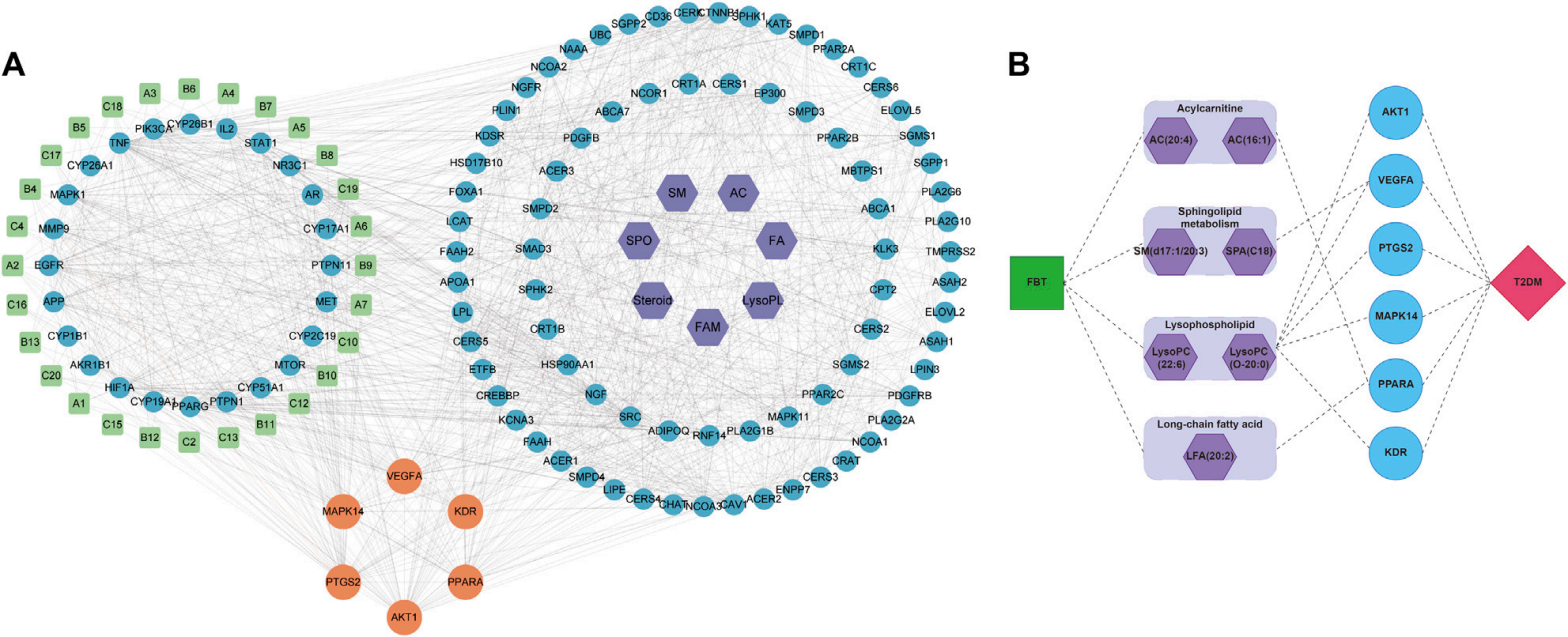
Combination of network pharmacology and global metabolomics. **(A)** Comprehensive PPI network analysis of “FBT-T2DM-discriminant metabolites.” **(B)** Connection of critical metabolites and targets.

The analytical process integrating network pharmacology and the results of global metabolomics are depicted in Figure 4. In this process, 243 potential targets of FBT and 990 potential targets of T2DM were obtained, as shown in Supplementary Figures S1, S2. The PPI network analysis of the common 62 targets was performed, and 24 critical target proteins were screened. Meanwhile, 88 predicted targets of discriminant metabolites were obtained, which were integrated with the above PPI network. These targets involved in both networks were screened out, as depicted in Figure 5A. The results focused on the six common targets, which included AKT1, VEGFA, PTGS2, MAPK14, PPARA, and KDR. The interactions of these critical metabolites and targets are depicted in Figure 5B.

These six targets were further imported into the Metascape database for GO and KEGG pathway enrichment analyses. The GO enrichment analysis showed that rich targets mainly involved the cell response to vascular endothelial, fatty acid metabolic processes, positive regulation of fat cell differentiation, regulation of inflammatory responses, and other biological processes, as depicted in Supplementary Figure S3. In addition, the KEGG pathway enrichment analysis showed that these targets would be involved in VEGF, MAPK, TNF, PI3K-Akt, and other signaling pathways, which were mainly related to the inflammation response, as depicted in Supplementary Figure S4. These enrichment results converged at the pathways related to inflammatory lipid metabolism, which needed further verification.

### 3.5 mRNA expression of screened target genes in the liver

**FIGURE 6:**
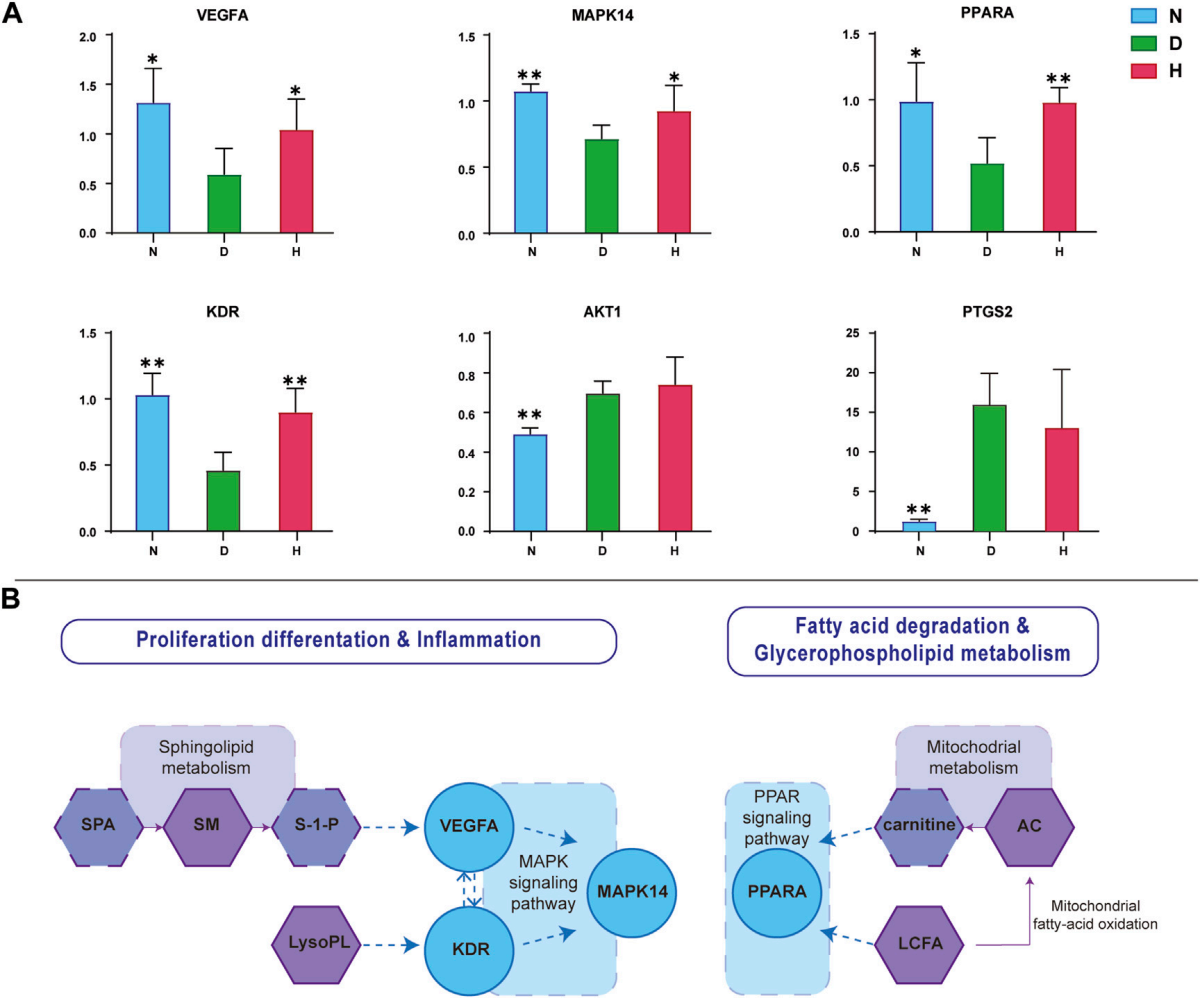
Verification of screened targets. **(A)** Relative levels of corresponding mRNA of six genes. **(B)** Involved pathways and metabolisms of target metabolites and genes. N, normal group; D, diabetic model group; H, high-dose FBT group.

To investigate the FBT impact on the mRNA expression of six screened target genes, the mRNA levels of AKT1, VEGFA, PTGS2, MAPK14, PPARA, and KDR in liver tissues were measured using RT-qPCR technology. The relative levels of mRNAs are displayed in Figure 6A. As a result, it was found that four gene levels, namely, VEGFA, MAPK14, PPARA, and KDR, in group N were significantly different from those in group D. In addition, their expressions all showed significant retracement after FBT-drinking treatment.

As shown in Figure 6B, the interactions of these metabolites and genes are mainly gathered at inflammation and lipid metabolism physiological processes. Sphingolipid metabolism and LysoPLs would affect the expression of KDR and VEGFA, respectively. VEGFA could biochemically interact with KDR. Meanwhile, VEGFA, KDR, and MAPK14 all converged on the MAPK signaling pathway involved in proliferation, differentiation, and inflammation. LCFA and AC would influence the expression of PPARA on the PPARA signaling pathway, which is mainly involved in glycerophospholipid metabolism and fatty acid degradation.

## 4 Discussion

The disorder of energy metabolism is a common symptom in T2DM patients (Emanuelli et al., 2014; Han et al., 2019). FBT is a functional food with multiple pharmacological effects on the regulation of metabolism. There was substantial evidence indicating that post-fermented tea had an obvious treatment effect on hyperlipidemia (Zhou et al., 2014; Ma et al., 2022). The treatment of FBT would contribute to an alteration in the liver metabolic profiles related to lipid metabolism and liver inflammation, which could restore dyslipidemia and liver injury (Zhou et al., 2022). Furthermore, as a type of bioactive component in FBT, theabrownin was indicated to effectively attenuate hyperlipidemia by inhibiting bile acids and intestinal lipid absorption in zebrafish (Xiao et al., 2023). Meanwhile, our previous research showed that the extract or fractions of FBT were helpful in relieving some typical symptoms of STZ-induced diabetes in mice (Xiang et al., 2020). Furthermore, we found that the extract of FBT had an α-glucosidase inhibitory effect *in vitro* (Xiang et al., 2021). The effective regulation of glycometabolism and lipometabolism would be the basis of prevention and therapy for T2DM.

In this study, global metabolomics and network pharmacology were combined to explore the hypoglycemic mechanism of FBT. Based on the result, the relevant metabolites were certain to be LysoPC, SM, LCFA, and AC. LysoPC is the key biomarker in the development of T2DM (Ahmad et al., 2021; He et al., 2021; Xu et al., 2021). According to the reports, the level of LysoPC would increase in the T2DM model (Sarosiek et al., 2016; Jiang et al., 2017), and its level was positively associated with oxidative stress (Bojko et al., 2021). In the kidneys of T2DM patients, the level of SMs played a role in mitochondrial function and inflammatory response (Miyamoto et al., 2016). The internal FAs were important to the structure of membrane lipids and then participated in the physiological processes of inflammation and insulin resistance (IR) (Wei et al., 2016). ACs were intermediate products of fatty acid oxidation (FAO), which could efficiently reflect the degree of FAO and mitochondrial oxidative stress (Koves et al., 2008; Libert et al., 2018). Both FAO and mitochondrial oxidative stress are critical factors in the development of IR and diabetes, so the variation in AC levels might be related to the conditions of obesity, IR, and diabetes (Mihalik et al., 2010; Mihalik et al., 2012; Bene et al., 2013).

After the determination of mRNA expression, VEGFA, KDR, MAPK14, and PPARA were verified to be the effective targets. In T2DM patients, the expression of soluble VEGF-receptor 1 was increased in monocytes under high-glucose conditions, which inhibited VEGF signaling (Makowski et al., 2021). VEGF resistance was a molecular concept that caused cellular dysfunction in diabetes mellitus (Tchaikovski et al., 2009). KDR encodes the kinase insert domain receptor protein, which is a corresponding receptor of VEGFA (Sharma et al., 2022). The expression of both VEGFA and KDR was reduced in gestational diabetes mellitus pregnancies compared to normal pregnancies (Meng et al., 2016). MAPK14 was the identified target gene in inhibition of glucose incorporation and triglyceride synthesis; its mRNA expression level was significantly reduced by overexpression of microRNA155 in HepG2 cells (Zhu et al., 2021). The MAPK family involves in a serial cascade that regulates the response to a variety of cellular signals, such as insulin signaling (Tang et al., 2014). In KEGG analysis, VEGFA, KDR, and MAPK14 all converge on the MAPK signaling pathway, which indicates the potential pharmacological mechanism of FBT. Likewise, PPARs participate in regulating several biological processes, including inflammation, glycolipid metabolism, and energy homeostasis (Gross et al., 2017). Increased expression of PPARA could protect pancreatic β-cell function and inhibit the development of T2DM (Lalloyer et al., 2006). PPARs are relevant therapeutic targets for drug design in the treatment of T2DM and dyslipidemia (Loza-Rodríguez et al., 2020). Based on these results, MAPK and PPAR signaling pathways might be the potential effective pathways of FBT drinking, which mainly point at inflammation and lipid metabolism in the development of diabetes mellitus.

Traditional network pharmacology analyses predict “drug-gene-disease” network based on biological information databases and platforms (Kim et al., 2020; Li et al., 2020), which lack the relevant experimental design. Therefore, the accuracy of analysis is extremely dependent on the objectivity of databases and algorithms. Meanwhile, the global metabolomic analysis could efficiently discover potential action pathways and biomarkers or monitor levels of discriminant metabolites through LC-QTOF-MS/MS technology. Integrating these two analytical approaches, the massive information provided offers a broad perspective incorporating bioinformatics and cheminformatics in this study. Due to their suitability for exploring the complex mechanisms of multi-components and multi-targets, it has become a popular tool for the pharmacological research on medicines with complex composition (Zhao et al., 2018; Wang et al., 2021).

## 5 Conclusion

In this study, global metabolomics and network pharmacology were combined to discover the potential target pathways and genes of FBT in the prevention of T2DM. Seven potential target metabolites and six potential target genes were singled out. Then, VEGFA, KDR, MAPK14, and PPARA were considered the target genes influenced by FBT-drinking treatment after the verification of RT-qPCR analysis. These target genes mainly converge on the MAPK and PPAR signaling pathway, which involved in the physiological processes of inflammation and lipid metabolism. Combining these results, it could be inferred that the hypoglycemic effect of FBT was closely related to its regulation of the expression of the above targets, and its intervention on inflammation and lipid metabolism. This research provided a meaningful exploration of the hypoglycemic pharmacological mechanism of FBT, which would be valuable for its further application and development in the future.

## Data Availability

The original contributions presented in the study are included in the article/Supplementary Material; further inquiries can be directed to the corresponding authors.

## Supplementary material

SUPPLEMENTARY FIGURE S1 Time axis of establishment of the T2DM model.

SUPPLEMENTARY FIGURE S2 Network analysis of FBT compound–target.

SUPPLEMENTARY FIGURE S3 Screening targets of type II diabetes mellitus.

SUPPLEMENTARY FIGURE S4 Result of the GO function enrichment analysis.

SUPPLEMENTARY FIGURE S5 Result of the KEGG pathway enrichment analysis.
