# Supplementary material for: Exploration of the hypoglycemic mechanism of Fuzhuan brick tea based on integrating global metabolomics and network pharmacology analysis
Source: Front Mol Biosci. 2024 Jan 18;10:1266156. doi: 10.3389/fmolb.2023.1266156 (PMC10830801; doi:10.3389/fmolb.2023.1266156)
Supplement: Supplementary file 1 [file Table1.DOCX]

**Table S1** The compositional data of basic diet of mice.

| Ingredient | Ratio (%) |
| --- | --- |
| Moisture | ≤10.0 |
| Crude protein | ≥18.0 |
| Crude fat | ≥4.0 |
| Crude fiber | ≤5.0 |
| Crude ash | ≤8.0 |
| Calcium | 1.0~1.8 |
| Phosphorus | 0.6~1.2 |

Source of materials: Protein source, soybean meal and fish meal; fat source, vegetable oil; fiber source, bran; carbohydrate, corn and wheat middlings.
