# Supplementary figures and images for: Exploration of the hypoglycemic mechanism of Fuzhuan brick tea based on integrating global metabolomics and network pharmacology analysis
Source: Front Mol Biosci. 2024 Jan 18;10:1266156. doi: 10.3389/fmolb.2023.1266156 (PMC10830801; doi:10.3389/fmolb.2023.1266156)

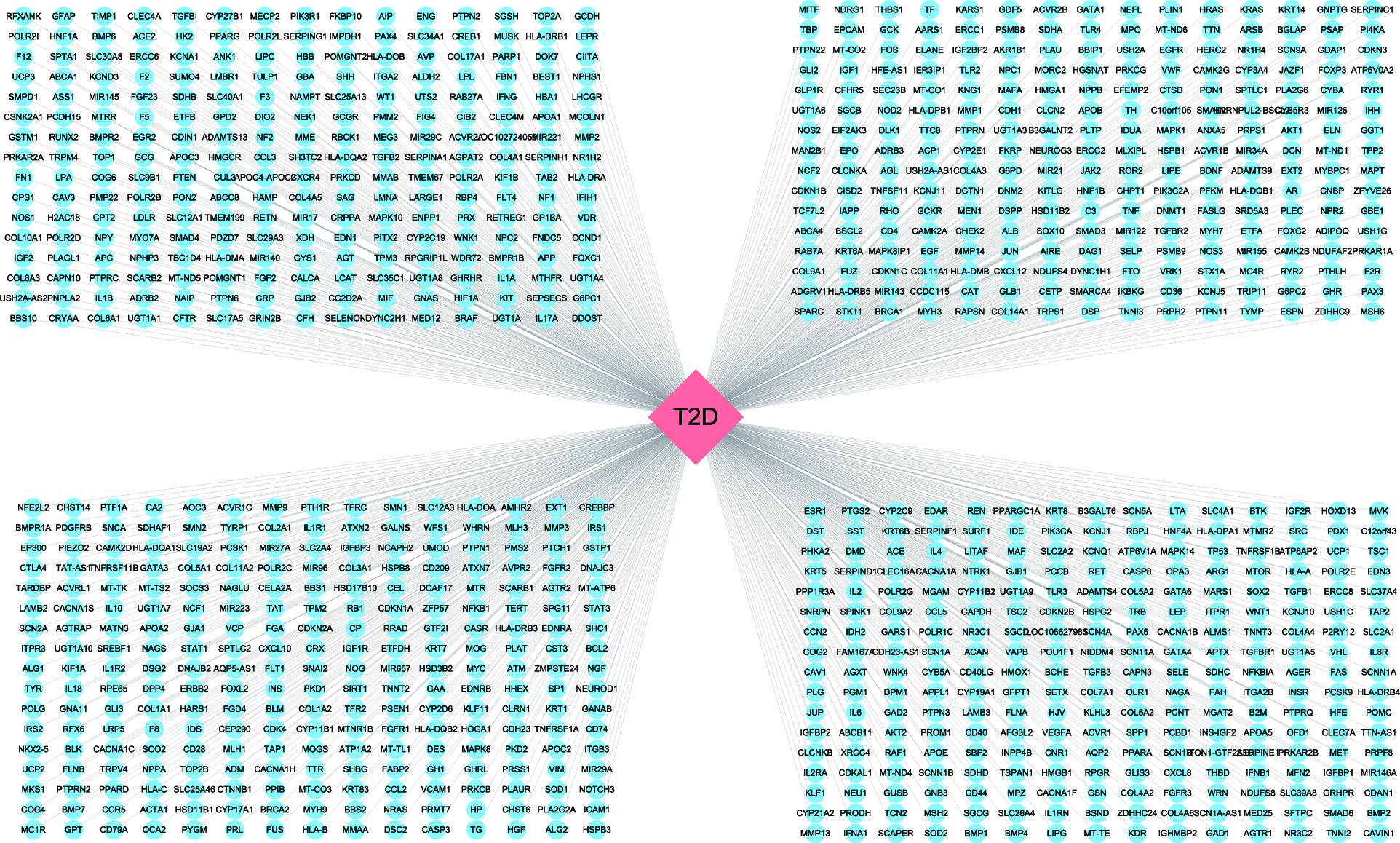

Supplement: Supplementary file 2 [file Image3.TIF]

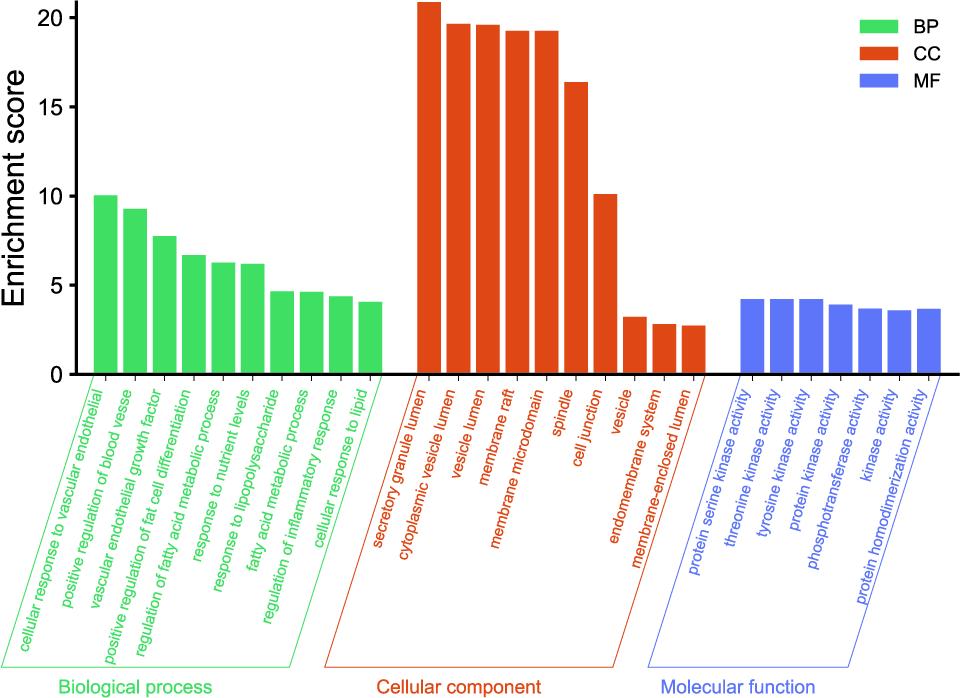

Supplement: Supplementary file 3 [file Image4.TIF]

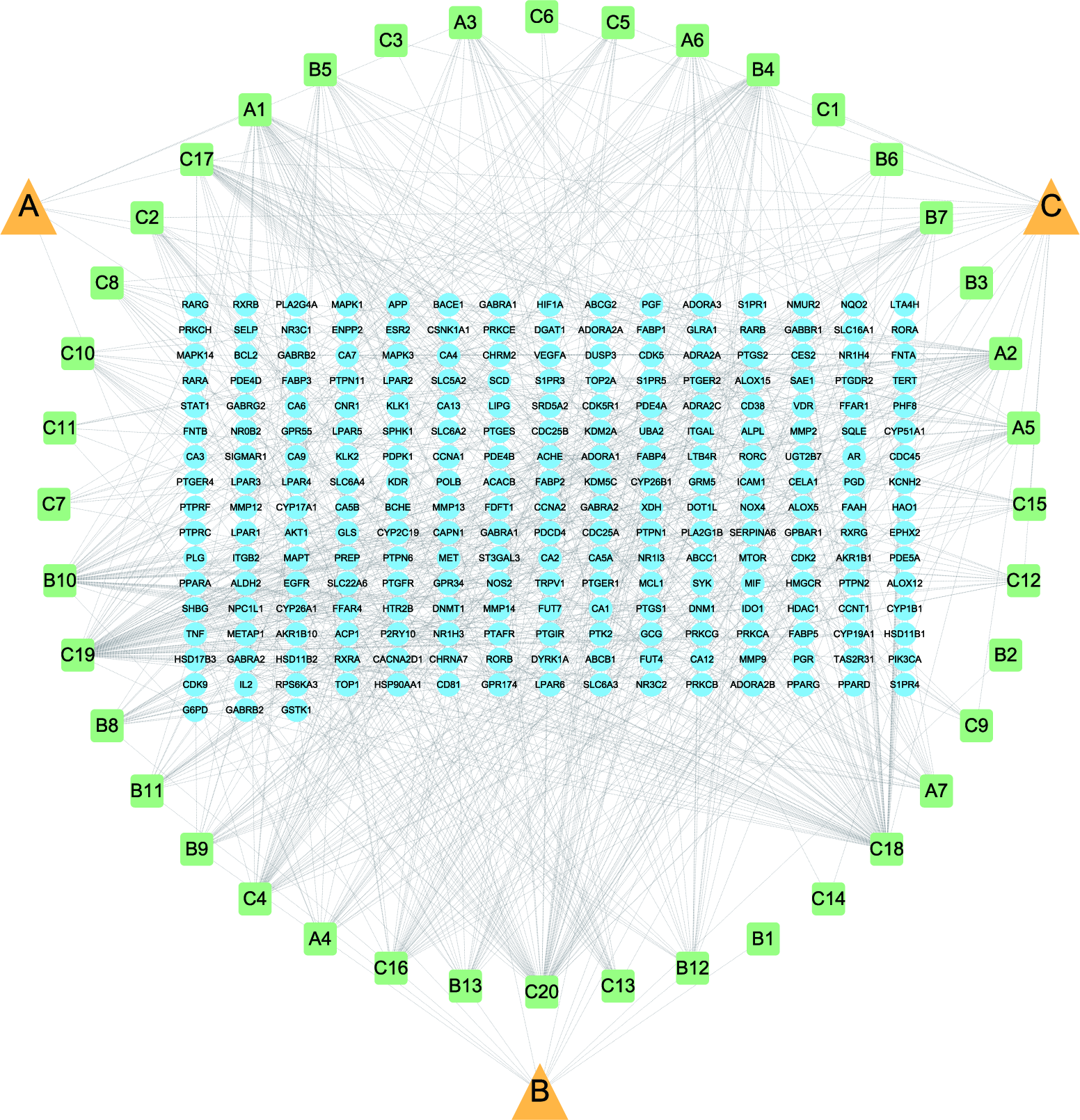

Supplement: Supplementary file 4 [file Image2.TIF]

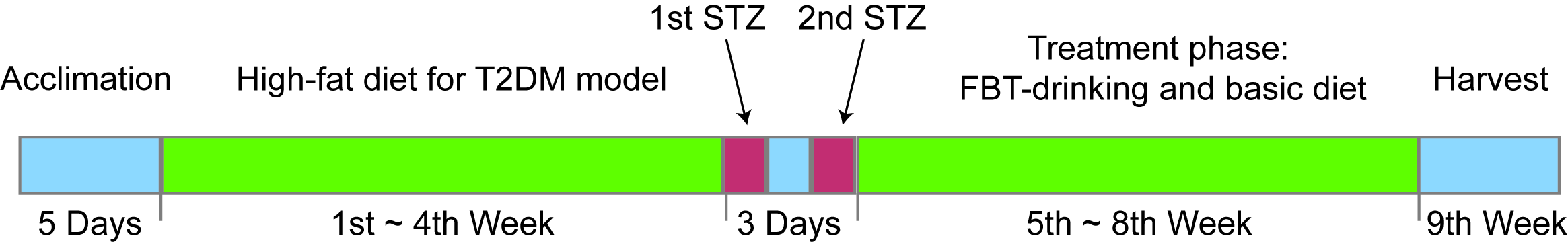

Supplement: Supplementary file 5 [file Image1.TIF]

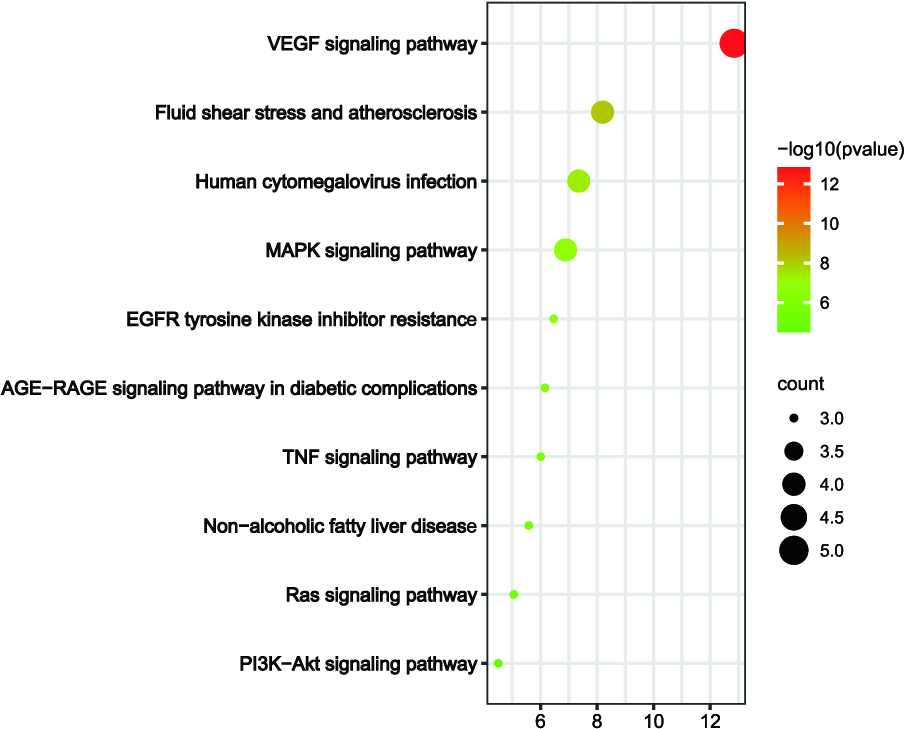

Supplement: Supplementary file 10 [file Image5.TIF]
