# Supplementary material for: Exploration of the hypoglycemic mechanism of Fuzhuan brick tea based on integrating global metabolomics and network pharmacology analysis
Source: Front Mol Biosci. 2024 Jan 18;10:1266156. doi: 10.3389/fmolb.2023.1266156 (PMC10830801; doi:10.3389/fmolb.2023.1266156)
Supplement: Supplementary file 6 [file Table2.DOCX]

**Table S2** The formulation of high-fat diet.

| Ingredient | Ratio (%) |
| --- | --- |
| Basic diet | 78.8 |
| Egg yolk | 10.0 |
| Lard oil | 10.0 |
| Cholesterol | 1.0 |
| Cholate | 0.2 |
