# Supplementary material for: Exploration of the hypoglycemic mechanism of Fuzhuan brick tea based on integrating global metabolomics and network pharmacology analysis
Source: Front Mol Biosci. 2024 Jan 18;10:1266156. doi: 10.3389/fmolb.2023.1266156 (PMC10830801; doi:10.3389/fmolb.2023.1266156)
Supplement: Supplementary file 7 [file Table5.docx]

**Table S5** The multiple mathematical statistical coefficients of excluded metabolites.

| No. | VIP^a^ | *U*-test^b^ | FDR^c^ | Fold change^d^ | Recovery^e^ | Direction of change^f^ |
| --- | --- | --- | --- | --- | --- | --- |
| C1 | 2.317 | 0.037 | 0.097 | 1.332 | Y | D |
| C2 | 2.203 | 0.631 | 0.752 | 1.308 | Y | D |
| C5 | 2.102 | 0.200 | 0.310 | 1.271 | Y | D |
| C6 | 2.069 | 0.262 | 0.387 | 0.843 | Y | U |
| C7 | 2.037 | 0.055 | 0.130 | 0.695 | Y | U |
| C8 | 1.995 | 0.337 | 0.454 | 1.337 | Y | D |
| C9 | 1.995 | 0.010 | 0.040 | 0.338 | Y | U |
| C10 | 1.993 | 0.037 | 0.097 | 1.598 | Y | D |
| C11 | 1.985 | 0.078 | 0.162 | 0.942 | N | D |
| C12 | 1.956 | 0.078 | 0.162 | 1.446 | Y | D |
| C13 | 1.943 | 1.000 | 1.000 | 0.881 | N | D |
| C14 | 1.930 | 0.749 | 0.774 | 0.942 | Y | U |
| C15 | 1.924 | 0.200 | 0.310 | 0.936 | Y | U |
| C16 | 1.886 | 0.016 | 0.056 | 2.160 | Y | D |
| C17 | 1.845 | 0.150 | 0.273 | 2.378 | Y | D |
| C19 | 1.710 | 0.749 | 0.774 | 0.958 | N | D |
| C20 | 1.699 | 0.337 | 0.454 | 1.118 | Y | D |
| C21 | 1.693 | 0.522 | 0.647 | 1.113 | N | U |
| C22 | 1.651 | 0.200 | 0.310 | 0.794 | Y | U |
| C23 | 1.644 | 0.004 | 0.040 | 1.356 | N | U |
| C24 | 1.608 | 0.749 | 0.774 | 0.923 | N | D |
| C26 | 1.583 | 0.749 | 0.774 | 0.924 | N | D |
| C28 | 1.550 | 0.423 | 0.547 | 1.007 | N | D |
| C29 | 1.547 | 0.109 | 0.212 | 1.608 | Y | D |
| C30 | 1.536 | 0.025 | 0.077 | 0.566 | Y | U |

^a^ VIPs were calculated by establishing OPLS-DA model with group N, D and H.

^b^ Raw *P* value of *U*-test was used for the intensity of metabolites in group N and D.

^c^ *P* value was adjusted by false discovery rate (FDR) correction was used as multiple correction.

^d^ Fold change was the ratio of the intensity of metabolites in group D and group N.

^e^ Recovery tend of the relative content of metabolite after FBT-drinking. Y indicated recovering after FBT-drinking; N indicated not recovering after FBT-drinking.

^f^ Direction of change after FBT-drinking. U indicated upregulate; D indicated downregulate.
