# Supplementary material for: Exploration of the hypoglycemic mechanism of Fuzhuan brick tea based on integrating global metabolomics and network pharmacology analysis
Source: Front Mol Biosci. 2024 Jan 18;10:1266156. doi: 10.3389/fmolb.2023.1266156 (PMC10830801; doi:10.3389/fmolb.2023.1266156)
Supplement: Supplementary file 8 [file Table3.DOCX]

**Table S3** Sequences of primers used in RT-qPCR.

| Gene |  | Sequences(5'-3') | Length(bp) |
| --- | --- | --- | --- |
| β-actin | Forward | GTGACGTTGACATCCGTAAAGA | 287 |
|  | Reverse | GTAACAGTCCGCCTAGAAGCAC |  |
| AKT1 | Forward | CTTCCTCCTCAAGAACGATGGC | 118 |
|  | Reverse | TGTCTTCATCAGCTGGCATTGT |  |
| VEGFA | Forward | GTAACGATGAAGCCCTGGAGTG | 244 |
|  | Reverse | TCACAGTGAACGCTCCAGGAT |  |
| PTGS2 | Forward | GTACCGCAAACGCTTCTCC | 235 |
|  | Reverse | TTGAGGAGAACAGATGGGATTT |  |
| MAPK14 | Forward | GACCGTTTCAGTCCATCATTCA | 191 |
|  | Reverse | CTGGCACTTCACGATGTTGTTC |  |
| PPARA | Forward | CACTACGGAGTTCACGCATGT | 166 |
|  | Reverse | GTGACATCCCGACAGACAGGC |  |
| KDR | Forward | GACGGATGATCAAGAGAAATAGAAC | 132 |
|  | Reverse | TGAGATACTTCACAGGGATTCGG |  |
