# Supplementary material for: Exploration of the hypoglycemic mechanism of Fuzhuan brick tea based on integrating global metabolomics and network pharmacology analysis
Source: Front Mol Biosci. 2024 Jan 18;10:1266156. doi: 10.3389/fmolb.2023.1266156 (PMC10830801; doi:10.3389/fmolb.2023.1266156)
Supplement: Supplementary file 9 [file Table4.docx]

**Table S4** Identification or characterization results of 31 differential metabolites.

| No. | RT (min) | Structures/name | Formula | Exact mass | Detected MS | MS/MS |
| --- | --- | --- | --- | --- | --- | --- |
| C1 | 15.63 | LysoPC (22:6) | C_30_H_50_NO_7_P | 568.3403 | [M+H]^+^ 568.3391 | 550.33, 258.11, 240.10, 184.07, 125.00, 104.10 |
| C2 | 13.86 | Amino-pentol | C_22_H_47_NO_5_ | 406.3532 | [M+H]^+^ 406.3536 | 332.29, 246.21, 218.17, 204.15, 174.14, 160.13, 146.12, 118.08 |
| C3 | 18.24 | FAM (22:1) | C_22_H_43_NO | 338.3423 | [M+H]^+^ 338.3438 | 321.32, 303.31, 279.31, 237.26, 223.24, 209.23, 195.21, 181.20, 139.15, 123.12 |
| C4 | 17.97 | AC (20:4) | C_27_H_45_NO_4_ | 448.3427 | [M+H]^+^ 448.3419 | 336.22, 310.21, 296.18, 230.14, 144.10 |
| C5 | 14.71 | LysoPI (20:4) | C_29_H_49_O_12_P | 621.3040 | [M-H]^-^ 619.2878 | 439.22, 303.23 |
| C6 | 14.24 | sphinganine (C17) | C_17_H_37_NO_2_ | 288.2903 | [M+H]^+^ 288.2908 | 239.24, 227.24, 197.23, 183.21, 169.20, 141.16 |
| C7 | 14.84 | sphinganine (C17) | C_17_H_37_NO_2_ | 288.2903 | [M+H]^+^ 288.2908 | 227.24, 169.19, 155.18 |
| C8 | 13.78 | Phytosphingosine (C18) | C_18_H_39_NO_3_ | 318.3008 | [M+H]^+^ 318.3019 | 300.29, 282.27, 239.23, 197.23, 183.21, 169.20, 155.18, 141.16, 127.15 |
| C9 | 17.46 | SM (d17:1/20:3) | C_42_H_79_N_2_O_8_P | 771.5652 | [M+H]^+^ 771.5610 | 184.07, 125.00, 104.11 |
| C10 | 15.94 | sphinganine (C18) | C_18_H_39_NO_2_ | 302.3059 | [M+H]^+^ 302.3060 | 284.30, 269.29, 239.24, 227.23, 197.22, 183.21, 169.19, 155.18, 127.15 |
| C11 | 14.01 | AC (13:0-OH) | C_20_H_39_NO_5_ | 374.2906 | [M+H]^+^ 374.2934 | 328.25, 300.21, 286.20, 230.16, 216.13, 188.09, 144.1017 |
| C12 | 15.94 | Phytosphingosine (C20) | C_20_H_43_NO_3_ | 346.3321 | [M+H]^+^ 346.3323 | 299.2930, 285.2829, 211.2443, 183.2142, 169.1930, 155.18, 127.15 |
| C13 | 18.12 | sphingosine (C18) | C_18_H_37_NO_2_ | 300.2903 | [M+H]^+^ 300.2886 | 265.25, 238.21, 197.22, 183.21, 169.20, 155.18, 141.17 |
| C14 | 19.30 | AC (16:0) | C_23_H_45_NO_4_ | 400.3427 | [M+H]^+^ 400.3412 | 356.28, 342.26, 258.17, 230.14, 144.10 |
| C15 | 14.36 | AC (12:0) | C_19_H_37_NO_4_ | 344.2801 | [M+H]^+^ 344.2752 | 285.20, 183.17, 144.10 |
| C16 | 17.10 | AC (16:1) | C_23_H_43_NO_4_ | 398.3270 | [M+H]^+^ 398.3253 | 235.28, 209.23, 162.11, 144.10 |
| C17 | 18.04 | PC (16:0/18:1) | C_42_H_82_NO_8_P | 760.5856 | [M+H]^+^ 760.5849 | 184.07, 125.00, 104.11 |
| C18 | 10.28 | Trihydroxypregn-ene-dione | C_21_H_30_O_5_ | 363.2171 | [M+H]^+^ 363.2205 | 303.19, 285.18, 261.19, 187.12, 163.11, 143.08 |
| C19 | 15.04 | LFA (18:3-OH) | C_18_H_30_O_3_ | 295.2273 | [M+H]^+^ 295.2252 | 277.22, 259.20, 187.15, 173.13, 159.19, 145.10, 131.09, 119.09 |
| C20 | 17.46 | Dioctyl hexanedioate | C_22_H_42_O_4_ | 371.3161 | [M+H]^+^ 371.3183 | 353.31, 225.18, 213.18, 199.17, 131.14 |
| C21 | 16.58 | MG (16:0) | C_19_H_38_O_4_ | 331.2848 | [M+H]^+^ 331.2860 | 313.28, 257.25, 239.24, 221.23 |
| C22 | 3.92 | AC (5:0) | C_12_H_23_NO_4_ | 246.1705 | [M+H]^+^ 246.1690 | 187.10, 144.10 |
| C23 | 16.83 | LysoPC (18:0) | C_26_H_54_NO_7_P | 524.3716 | [M+H]^+^ 524.3730 | 258.10, 184.07, 125.00, 104.11 |
| C24 | 15.09 | LysoPI (20:3) | C_29_H_51_O_12_P | 623.3196 | [M-H]^-^ 621.3022 | 441.24, 305.24 |
| C25 | 18.29 | LysoPC (O-20:0) | C_28_H_60_NO_6_P | 538.4237 | [M+H]^+^ 538.4201 | 184.07, 124.10, 104.11 |
| C26 | 13.75 | sphinganine (C17) | C_17_H_37_NO_2_ | 288.2903 | [M+H]^+^ 288.2908 | 227.24, 197.23, 169.19, 155.18 |
| C27 | 17.56 | LFA (20:2) | C_20_H_36_O_2_ | 309.2794 | [M+H]^+^ 309.2800 | 263.27, 249.25, 207.21, 163.15, 139.15 |
| C28 | 17.83 | AC (17:0) | C_24_H_47_NO_4_ | 414.3583 | [M+H]^+^ 414.3578 | 355.29, 183.21, 144.10 |
| C29 | 17.63 | FAM (22:2) | C_22_H_41_NO | 336.3266 | [M+H]^+^ 336.3256 | 277.29, 263.27, 235.24, 207.21, 165.17 |
| C30 | 17.21 | AC (16:1) | C_23_H_43_NO_4_ | 398.3270 | [M+H]^+^ 398.3253 | 209.22, 195.21, 169.20, 144.10 |
| C31 | 10.19 | Acetoxy-androstene-dione | C_21_H_28_O_4_ | 345.2066 | [M+H]^+^ 345.2098 | 345.21, 317.20, 299.17, 285.18, 233.11, 217.09, 161.10, 123.08 |

LysoPC, lysophosphatidylcholine; FAM, fatty amide; AC, acylcarnitine; LysoPI, lysophosphatidylinositol; SM, sphingomyelin; PC, phosphatidylcholine; LFA, long-chain fatty acid; MG, monoacylglyceride.

Due to limits of UPLC-QTOF-MS/MS, locations of double bonds in fatty acids, the hydroxyls in flavonoids or the substituent groups can not be determined.
